# Optimization of Injection-Molding Process for Thin-Walled Polypropylene Part Using Artificial Neural Network and Taguchi Techniques

**DOI:** 10.3390/polym13234158

**Published:** 2021-11-28

**Authors:** Mehdi Moayyedian, Ali Dinc, Ali Mamedov

**Affiliations:** College of Engineering and Technology, American University of the Middle East, Kuwait; mehdi.moayyedian@aum.edu.kw (M.M.); ali.dinc@aum.edu.kw (A.D.)

**Keywords:** injection molding, shrinkage, warpage, short shot, Taguchi, artificial neural network

## Abstract

Plastics are commonly used engineering materials, and the injection-molding process is well known as an efficient and economic manufacturing technique for producing plastic parts with various shapes and complex geometries. However, there are certain manufacturing defects related to the injection-molding process, such as short shot, shrinkage, and warpage. This research aims to find optimum process parameters for high-quality end products with minimum defect possibility. The Artificial Neural Network and Taguchi Techniques are used to find a set of optimal process parameters. The Analytic Hierarchy Process is used to calculate the weight of each defect in the proposed thin-walled part. The Finite Element Analysis (FEA) using SolidWorks plastics is used to simulate the injection-molding process for polypropylene parts and validate the proposed optimal set of process parameters. Results showed the best end-product quality was achieved at a filling time of 1 s, cooling time of 3 s, pressure-holding time of 3 s, and melt temperature of 230 °C. The end-product quality was mostly influenced by filling time, followed by the pressure-holding time. It was found that the margin of error for the proposed optimization methods was 1.5%, resulting from any uncontrollable parameters affecting the injection-molding process.

## 1. Introduction

Plastics offer a wide range of advanced mechanical properties such as high strength-to-weight ratio, flexibility, corrosion resistance, transparency, etc., which make them irreplaceable materials in various engineering fields such as automobile and aerospace industries, electronics, and biomedical industries. For instance, plastics are found in a range of applications, such as in aircraft windshields, automobile windows, medical and dental equipment, food and beverage packaging, and petrol-chemical storage. Injection-molding processes are used for manufacturing plastic end-products for most of the aforementioned applications. Injection-molding processes can be described in three simple phases: (1) filling phase, where the molten polymer is injected into a mold cavity with the desired shape; (2) packaging phase, where high packing pressure is applied to ensure proper filling of the mold cavity; and (3) cooling phase, where the temperature of the mold is decreased, and the polymer solidifies. The quality of an end-product in injection molding is the result of a complex combination of the process parameters, part/mold design, and material used. In this paper, three common defects which reduce the quality of end-products are evaluated: (1) short shot, (2) shrinkage rate, and (3) warpage. Warpage is defined as a serious defect in injection-molded parts, especially the thin-walled products [1]. Many researchers tried to minimize warpage defects using different techniques. Kurtaran and Erzurumlu [2] used response surface methodology and genetic algorithm to achieve the minimum warpage. Gao et al. [3] used the Kriging surrogate model trained by numerical simulation to acquire more stable product quality compared to traditional methods. Kim and Lee [4] used the modified complex method and obtained warpage reduction over 70% by controlling wall thickness and process parameters. Yin et al. [5] used backpropagation neural network modeling for precise prediction of warpage in plastic parts. Injection molding shrinkage deals with dimensional differences between a molded part and the cavity. The shrinkage behavior of a molded plastic part plays an important role in determining the final dimensions of the part [6]. Lotti et al. [7] used an Artificial Neural Networks approach to predict the shrinkage of injection-molded plastic plaques. Tang et al. [8] and Hassan et al. [9] studied a cooling system design in terms of cooling channel size and location for multi-cavity injection molds to ensure uniform solidification inside a mold cavity, which would prevent shrinkage. A short shot is the incomplete filling of a mold cavity, which results in the production of an incomplete part. In general, a short shot occurs when insufficient material is injected into the mold or flow freezes before the mold cavity is fully filled [10]. It is caused by different factors such as the wrong plastic material selection, incorrect processing parameters, incorrect mold design, and part design [11]. Moayyedian et al. [12] mentioned that the cross-sectional shape of a gate or runner leads to short shots at the filling stage. The influence of runner/gate design on the quality of an injected part was also investigated by Tsai [13], who placed a rectangular flow restrictor within the tertiary runner of a precision optical lens mold to achieve uniform melt temperature distribution in the runner channel and reduce the thermal residual stress and warpage of injection-molded parts. Shen et al. [14] investigated optimal gate design for thin-walled injection molding and noticed that gate design affects the shear rate, which in turn increases the material’s temperature. The higher temperature can reduce the viscosity of melted plastic so that the melted plastic can fill into the cavity easily. Kim et al. [15] used numerical analysis to investigate polymer flow patterns for different gate locations, and results showed that wrong positioning of the gate prevented flow to the other side of the part and resulted in short shots.

All presented research tells us that if molding process parameters can be adjusted in an intelligent way, the quality and mechanical performance of the end-product can be improved. Different from previously presented studies that analyze particular defects, this paper presents a novel approach to the quality evaluation of the injected part. In this paper, the Artificial Neural Network and Taguchi Techniques are used to find a set of optimal process parameters that will result in a part with minimum possible short shot, shrinkage rate, and warpage. The Analytic Hierarchy Process is used to calculate the weight of each defect in the proposed thin-walled part design. The Taguchi method is used to find an optimal set of five different geometric and process parameters in three different levels that will result in the highest end-product quality. Finite Element Analysis (FEA) using SolidWorks plastics is used to simulate injection-molding process experiments and validate a proposed optimal set of process parameters.

## 2. Proposed Methodology

### 2.1. Problem Description

There are different internal and external defects in injection-molding technology that evaluate the quality of injected parts, such as sink mark, shrinkage, warpage, weld line, and short shot. In this paper, three common defects which reduce the quality of injected parts were chosen: short shot possibility, shrinkage rate, and warpage. The possibility of having the selected defects is related to different geometrical and process parameters.

### 2.2. Weight Calculation for the Selected Defects via AHP

The initial weight of each plastic defect was calculated via the Analytic Hierarchy Process, as shown in Table 1. Short shot had the highest weight followed by warpage and shrinkage, respectively.

### 2.3. Taguchi Orthogonal Array

The Taguchi method has been employed over the years to improve products and manufacturing processes. It is a powerful and effective method to solve the quality problems of products [16,17]. The objective of this paper is to combine the Taguchi method with simulation tools, namely SolidWorks plastics, to reduce the percentage of different internal and external defects in injection molding. Five different geometric and process parameters in three different levels are selected, as shown in Table 2. Selecting the parameters is based on the literature review, with a high percentage of contribution through the injection process for the evaluation of the selected defects. Additionally, based on the number of parameters and number of levels, an L18 orthogonal array is selected, as shown in Table 3.

## 3. Simulation

**Figure 1:**
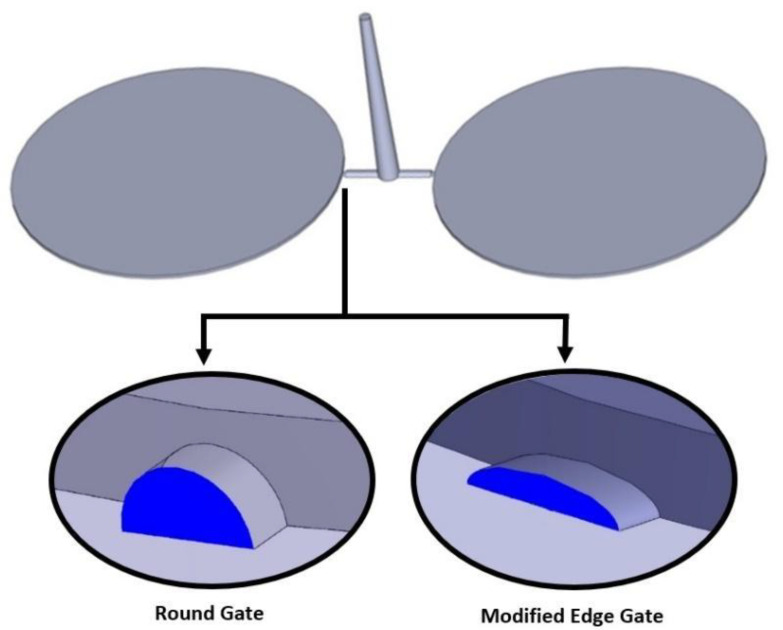
The 3D design of plastic part with sprue, runner and two types of gate system.

Two circular parts with 100 mm diameter and 1 mm thickness are designed using SolidWorks, as shown in Figure 1. Sprue, runner, and gate have also been calculated and designed with reference to the geometry, the dimension of the selected design, and the selected material. To evaluate the selected defects in a critical condition, 1 mm thickness and round shape parts are selected to avoid having any extraneous variables such as the effect of corners or busses on the flow of molten plastic through the injection process. Since the gate type leads to short shot and shrinkage, two different gates have been selected as shown in Figure 1, namely round gate and modified edge gate [11].

**Figure 2:**
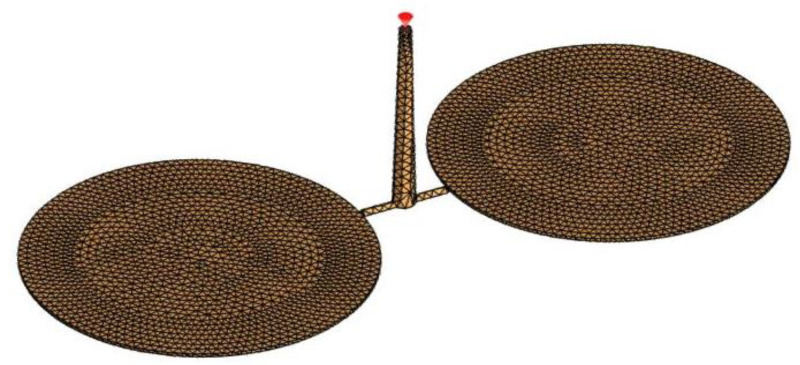
Finite element analysis for 3D part design.

For the flow analysis, SolidWorks plastic is applied, and Finite Element Analysis (FEA) is conducted with shell (triangle) mesh with element thickness of 1 mm, as shown in Figure 2. Polypropylene (P.P.) material was selected for the analyses. The glass transition temperature of the material (T_g_) is 135 °C, and the viscosity model is presented in Table 4. The mesh refinement is implemented with element size of 0.3 mm for sprue and runner and 0.2 mm for the gate. To avoid having any extraneous variable affecting the result for the selected plastic defects, one of the input parameters, which needs to be set through the simulation process, is mold temperature. Hence, the mold temperature is 50 °C as one of the constants. The mesh details are tabulated in Table 5 in the following:

### 3.1. Experimental Setup

In this paper, polypropylene was chosen as the injected material for the injection of two circular plates. Material characteristics are listed in Table 4. For the fabrication process, computer numerical control (CNC) milling machine, grinding machine, and drilling machine are used to produce the main components of mold tools, namely top clamping plate, core and cavity plates, side plates, and bottom clamping plate. The selected injection machine was the Poolad-Bch series with maximum inlet pressure of 100 MPa. The details of the process parameters are presented below in Table 6.

A two-plate mold with two cavities and one parting line with runner, gate, and sprue but without ejector system is chosen, and the selected material for the fabrication of core and cavity is steel CK45 with surface hardness 56 HRC.

### 3.2. Simulation Results

**Figure 3:**
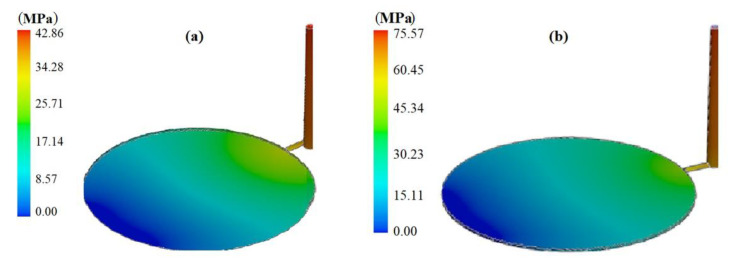
Injected parts with (**a**) minimum possibility of short shot and (**b**) maximum possibility of short shot.

The analysis of short shot possibility is implemented using SolidWorks plastic. Short shots happen far from the gate locations or on thin wall products. They also happen as a result of insufficient venting [18]. In analyzing the short shot possibility (the ratio of simulated inlet pressure to maximum inlet pressure), different factors are taken into consideration to diagnose short shot before it occurs [19]. The minimum level of short shot possibility is related to experiment number 17, as shown in Figure 3a, and the maximum short shot possibility is related to experiment number 1, as shown in Figure 3b. Any increase in filling time, part cooling time, and melt temperature will decrease the short shot possibility, as shown in experiment number 17. In contrast, in experiment number 1, filling time, part cooling time, and melt temperature are at their minimum levels, which leads to a high level of short shot possibility.

**Figure 4:**
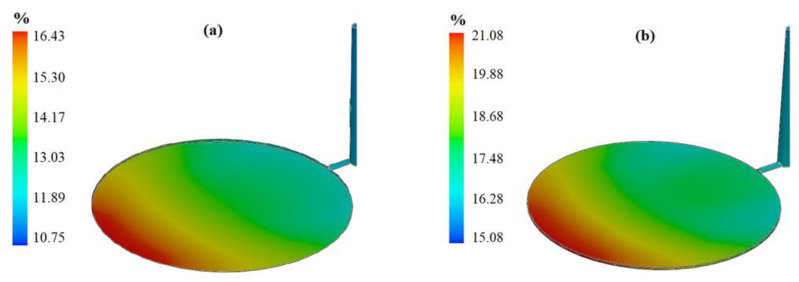
Injected parts with (**a**) minimum shrinkage and (**b**) maximum shrinkage.

The second analysis was shrinkage analysis. The difference between the linear dimensions of the cavity and the injected parts at room temperature will evaluate the shrinkage rate [20]. Experiment number 14 represents the minimum shrinkage rate, and experiment number 5 represents the highest shrinkage rate, as shown in Figure 4a,b, respectively. With reference to the simulation result, it can be concluded that when the melt temperature increases, the shrinkage rate increases.

**Figure 5:**
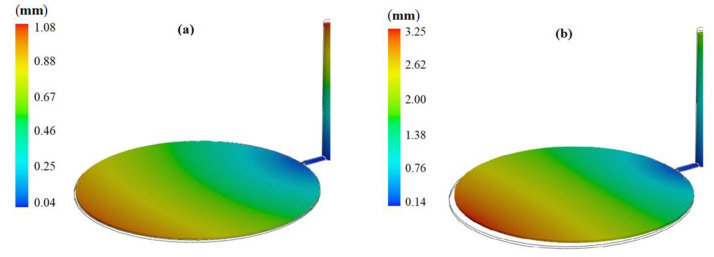
Injected parts with (**a**) minimum warpage and (**b**) maximum warpage.

The last defect analysis for quality purposes is warpage, which refers to a distortion of the original design of the injected parts because of different shrinkage rates in different parts of the injected part [18]. With reference to the simulation result, the minimum warpage is related to experiment 6, and the maximum warpage is related to experiment 9, as shown in Figure 5a,b, respectively. Hence, any increase in melt temperature and filling time based on Table 3 will result in an increase in the warpage percentage.

Based on the L18 orthogonal array of Taguchi 18, experiments have been conducted with different settings using SolidWorks plastics, and the defect values were tabulated, as shown in Table 7. Maximum and minimum values for each defect type are highlighted together with corresponding experiment numbers.

Weight calculation for the selected defects is implemented, as shown in Table 8, and the sum of the defect’s value for individual experiments has been calculated. In a similar way, normalized maximum and minimum values for each defect type are highlighted together with corresponding experiment numbers.

Since the objective of this study is to minimize different defects in injection molding, the smaller-the better-quality characteristic has been chosen, which is defined by Equations (1) and (2) [21]:

(1) $$\frac{S}{N}=-10\log\left(MSD\right)$$

(2) $$MSD=\frac{1}{N}\overset{n}{\underset{i=1}{\sum }}y_{i}^{2}$$

where *y_i_* is the total value of the selected defects for different experiments and *N* is the total number of data points. Signal-to-noise ratio calculation has been conducted and tabulated in Table 9. The next step was to determine the response table of Taguchi to find the most significant parameters from the selected parameters and their optimum levels.

With reference to Table 10, the optimum level is the highest value of each parameter. Hence, the best combination is gate type at level 1, filling time at level 2, cooling time at level 2, pressure-holding time at level 3, and melt temperature at level 3. 

**Figure 6:**
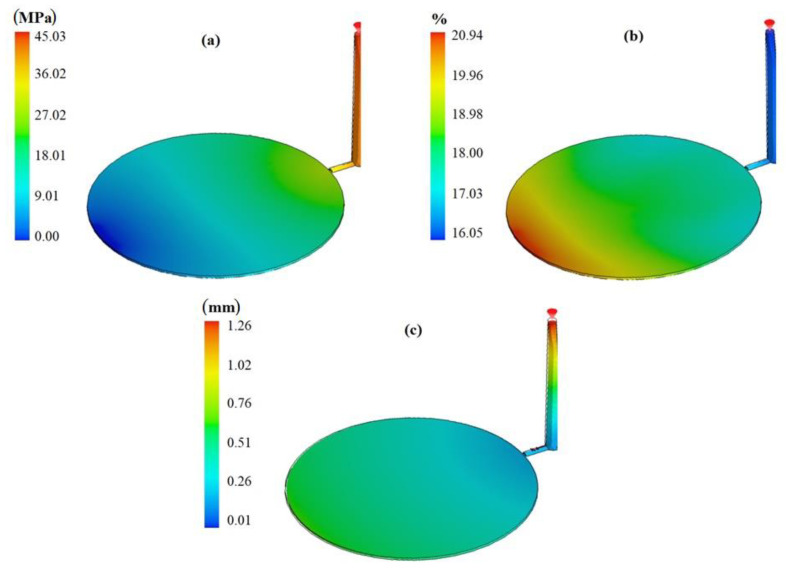
Defect values based on the optimum level (**a**) short shot, (**b**) shrinkage, (**c**) warpage.

The next step was to run the simulation based on the optimum level to evaluate the individual defect values and the sum of the selected defects. Based on the simulation results as shown in Figure 6, the optimum defects values are tabulated in Table 11, as shown in the following:

With reference to Table 11, running the simulation with optimum level of the minimum defects rate shows that the used optimization tool gives good results for the injection-molding process. The proposed methodology was experimentally validated by Moayyedian [21].

The final step was to apply analysis of variance to determine the percentage of contribution for individual parameters. The percentage of contribution can be calculated as follows [17]:

1. Degree of freedom: The total degree of freedom ($df_{T}$ ), the degree of freedom of factor A ($df_{A}$), and the degree of freedom for error variance ($df_{E}$) are as follows:

(3) $$df_{T}=\left(N-1\right)$$

(4) $$\;df_{A}=\left(K_{A}-1\right)$$

(5) $$df_{E}=\left(df_{T}-\sum^{}df_{factor}\right)$$

where *N* is the total number of experiments.

2. Sum of squares: The sum of the square of factor A ($SS_{A}$ ), the total sum of square ($SS_{T}$) and the sum of the square for error variance ($SS_{E}$) are calculated as follows:

(6) $$SS_{A}=\overset{K_{A}}{\underset{i=1}{\sum }}\left(\frac{A_{i}^{2}}{n_{A_{i}}}\right)-\frac{{\left(\sum_{i=1}^{N}x_{i}\right)}^{2}}{N}$$

(7) $$SS_{T}=\overset{N}{\underset{i=1}{\sum }}x_{i}{}^{2}-\frac{{\left(\sum_{i=1}^{N}x_{i}\right)}^{2}}{N}$$

(8) $$SS_{E}=\left(SS_{T}-\sum^{}SS_{factor}\right)$$

where $x_{i}$ is a value at level (1, 2, … *N*),$\;n_{A_{i}}$ is the number of levels and $A_{i}$ is a value at level *i* of factor *A*.

3. Percentage contribution: the percentage contribution of factor A is calculated using the following Equation:


(9) $$PC_{A}=\frac{SS_{A}}{SS_{T}}\times 100\%$$

The percentage of contribution for the selected factors is tabulated in Table 12 in the following:

By determining the optimum level and the significant parameters reducing the total defects value, the next step was to evaluate the percentage of contribution, based on Equations 3 to 9. The percentage of contribution for individual parameters can be achieved by employing an ANOVA. The largest value of contribution indicates the most significant factor affecting the system’s performance. It can be concluded that the filling time has the highest percentage of contribution (42.8%), followed by pressure-holding time (26.6%).

## 4. Artificial Neural Network Model

Artificial Neural Network (ANN) is a modeling tool that has a particular ability to learn and generate functions from training series. ANNs establish the relationships between inputs and outputs using particular transfer functions. In a series of training operations, these are used to alter the values of the biases and weights. ANNs are made up of neurons, which are small, linked processors. Weighted linkages connect the neurons, allowing messages to travel between them. Each neuron receives many inputs according to their connection weights from other neurons and creates a single output that may propagate to several additional neurons [22,23]. The backpropagation learning algorithm has been the most widely employed approach in engineering applications among the numerous types of ANNs that exist. The Levenberg–Marquardt backpropagation training algorithm was selected for this study.

Two phases are involved in the ANN modeling process. The first phase is to train the network, and the second is to test it using data that were not utilized in the training process. It is critical that the network obtains all of the information it needs to learn in the form of a data set. When the network reads each pattern, it utilizes the input data to generate an output, which is then compared to the training pattern. If there is a discrepancy, the connection weights are adjusted in a way that reduces the error. If the error is still more than the maximum acceptable tolerance after the network has gone through all of the input patterns, the ANN goes through all of the input patterns again until all of the errors are within the necessary tolerance [24].

**Figure 7:**
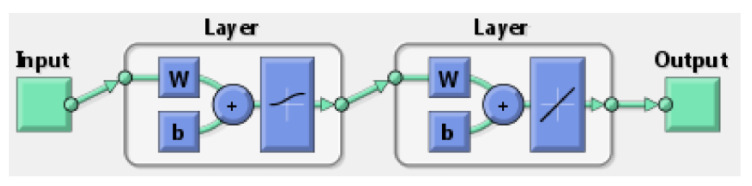
Neural network.

ANN was employed for the intended range of four inputs (filling time, part-cooling time, pressure-holding time, and melt temp). Figure 7 shows this model with the appropriate neuron numbers in the hidden and output layers. A normalized and weighted output was used, which represents the associated values of three outputs (short shot, shrinkage rate, warpage) as given in Table 8 earlier. The backpropagation (BP) training technique was used to create this neural network unit because it has the capacity to forecast values in between learning values and make interpolations between learning-curves data. This was accomplished using the appropriate amount of network layers and neurons at minimum error.

**Figure 8:**
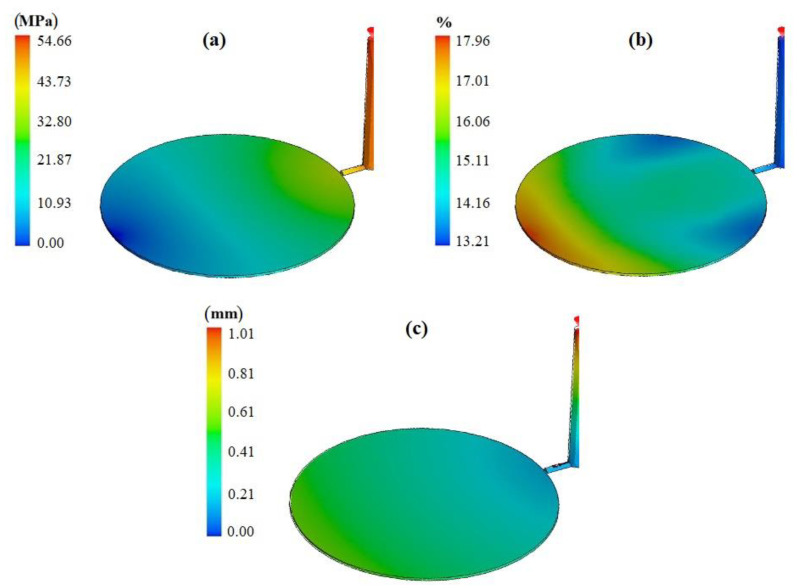
Defects value based on the optimum level (**a**) short shot, (**b**) shrinkage, (**c**) warpage.

After obtaining the ANN model, it was coupled with an optimization algorithm in the computer code. The objective was to obtain the minimum “output” value. After convergence of optimization with the ANN model, the best output value was obtained with the following parameters: filling time at level 3, cooling time at level 1, pressure-holding time at level 3, and melt temperature at level 2. The result calculated by ANN optimization was 0.2542. In order to validate the “predicted best case scenario”, an additional SolidWorks simulation was performed to check the result for this particular case, as shown in Figure 8. SolidWorks simulation gave an output of 0.2461, as shown in Table 13, which is close to the ANN prediction and better than all trials in Table 8. In other words, SolidWorks simulation validated that the parameter configuration proposed by the ANN results in the best output compared to all previous trials.

The comparison of predictions by the Taguchi Method and ANN model was made based on the SolidWorks simulation results of both cases. The simulation result for the parameters predicted with the Taguchi method given in Table 11 was **0.2499,** and the simulation result for parameters predicted with the ANN method given in Table 13 was **0.2461**. It shows that the Taguchi Method and ANN model predictions were successful in estimating the best cases (minimum output) and were close in value. The margin of error was calculated, as shown in Equation (10):

(10) $$\mathrm{Margin}\;\mathrm{of}\;\mathrm{error}\;\%=(\frac{\mathrm{Taguchi}\;\mathrm{method}-\mathrm{ANN}}{\mathrm{ANN}})\;\times \;100$$

With reference to Equation (10), the margin of error for the Taguchi method and ANN is equal to 1.5%, which is within the acceptable range in engineering fields.

## 5. Conclusions

The combination of simulation with DOE was a useful approach to find the significant parameters that lead to short shot, warpage, and shrinkage of the injected part. Different processes and geometric parameters were selected for the proposed solution. Based on the selected orthogonal array of Taguchi, 18 experiments were conducted via SolidWorks plastics and the finite element method (FEM) to determine the optimum level of the selected parameters to minimize different internal and external defects. The signal-to-noise ratio (S/N) was an effective tool to determine the optimum level of each parameter, and an ANOVA was used for determining the percentage of contribution. It can be concluded that filling time had the highest percentage of contribution (42.8%), followed by pressure-holding time (26.6%). 

The ANN was applied to determine the optimum levels of different parameters to minimize the selected defects. The normalized output value based on the ANN model and FEM simulations was 0.2542 and 0.2461, respectively, which were very close to each other. The normalized simulation output values of the ANN model and Taguchi method were 0.2461 and 0.2499, respectively. The margin of error percentage between the ANN model and Taguchi method was equal to 1.5%, which demonstrated the robustness of the proposed method and the compatibility of the selected tools. It can be concluded that the predicted model with minimum defects had been selected, which was the ANN model. The selected optimum model was to have filling time at 1 s, cooling time at 3 s, pressure-holding time at 3 s, and melt temperature at 230 °C. The optimum level of the selected parameters based on the ANN model was very realistic, resulting in the lower temperature to avoid having any other defects related to high temperature and lower part-cooling time to reduce the injection time. Further research in this direction will provide more comprehensive guidelines for designers by considering other processes and geometric parameters which increase different defect rates in injection molding.

## Figures and Tables

**Table 1:** Calculation of initial weights.

	S1	S2	S3	Initial Weight
Step weight	0.5	0.2	0.3	
Short shot	1			0.5
Shrinkage rate		1		0.2
warpage			1	0.3

**Table 2:** Geometric and process parameters in three levels.

Parameters	Level 1	Level 2	Level 3
Gate design, A	1	2	-
Filling time, B (s)	0.2	0.6	1
Part cooling time, C (s)	3	3.9	5
Pressure holding time, D (s)	1	2	3
Melt temperature, E (°C)	200	230	280

**Table 3:** L18 orthogonal array.

Experiment	A	B	C	D	E
1	1	1	1	1	1
2	1	1	2	2	2
3	1	1	3	3	3
4	1	2	1	1	2
5	1	2	2	2	3
6	1	2	3	3	1
7	1	3	1	2	1
8	1	3	2	3	2
9	1	3	3	1	3
10	2	1	1	3	3
11	2	1	2	1	1
12	2	1	3	2	2
13	2	2	1	2	3
14	2	2	2	3	1
15	2	2	3	1	2
16	2	3	1	3	2
17	2	3	2	1	3
18	2	3	3	2	1

**Table 4:** Modified cross-model for viscosity.

D1 (Pa)	D2 (K)	D3	A1	A2 (K)	τ (Pa)	n
4.44489 × 10^14^	263.15	0	32.7	52.6	26,260	0.272

**Table 5:** FEA simulation and mesh parameters.

Mesh Type	Meshing Method	Triangle Size (mm)	Total Node of Surface Mesh	Total Elements of Surface Mesh	Mesh Size for Runner (mm)	Mesh Size for Gate (mm)
Shell Mesh	Manual	2.76	5624	11,244	0.3	0.2

**Table 6:** Process parameters.

Melt Temperature	Max Melt Temperature	Min Melt Temperature	Mold Temperature	Melt Flow Rate	Max Shear Stress
230 °C	280 °C	200 °C	50 °C	20 cm^3^ /10 min	250 kPa

**Table 7:** Defects determination of 18 experiments based on SolidWorks plastics results.

Trial Number	Short Shot	Shrinkage Rate	Warpage
1	75.57	16.45	2.43
2	64.72	18.24	1.73
3	52.65	21.08	1.54
4	55.34	18.24	2.64
5	44.8	21.08	1.94
6	64.57	16.43	1.08
7	62.4	16.43	1.3
8	53.49	18.23	1.25
9	43.27	21.04	3.25
10	72.27	16.44	2.48
11	72.08	16.44	2.48
12	62.17	18.24	1.77
13	43.81	21.08	1.83
14	62.72	16.43	1.08
15	53.98	18.24	2.78
16	52.76	18.23	2.78
17	42.86	21.04	2.78
18	61.18	16.43	2.78

**Table 8:** Normalized defect based on initial weight calculation.

Trial Number	Short Shot	Shrinkage Rate	Warpage	Sum
1	0.500	0.001	0.188	0.69
2	0.334	0.078	0.090	0.50
3	0.150	0.200	0.064	0.41
4	0.191	0.078	0.217	0.49
5	0.030	0.200	0.119	0.35
6	0.332	0.000	0.000	0.33
7	0.299	0.000	0.031	0.33
8	0.162	0.077	0.024	0.26
9	0.006	0.198	0.300	0.50
10	0.450	0.000	0.194	0.64
11	0.447	0.000	0.194	0.64
12	0.295	0.078	0.096	0.47
13	0.015	0.200	0.104	0.32
14	0.304	0.000	0.000	0.30
15	0.170	0.078	0.236	0.48
16	0.151	0.077	0.236	0.46
17	0.000	0.198	0.236	0.43
18	0.280	0.000	0.236	0.52

**Table 9:** Signal-to-noise ratio for the smaller-the-better quality characteristics.

Experiment	A	B	C	D	E	S/N
1	1	1	1	1	1	3.24
2	1	1	2	2	2	5.98
3	1	1	3	3	3	7.67
4	1	2	1	1	2	6.28
5	1	2	2	2	3	9.14
6	1	2	3	3	1	9.58
7	1	3	1	2	1	9.65
8	1	3	2	3	2	11.58
9	1	3	3	1	3	5.94
10	2	1	1	3	3	3.82
11	2	1	2	1	1	3.86
12	2	1	3	2	2	6.58
13	2	2	1	2	3	9.93
14	2	2	2	3	1	10.35
15	2	2	3	1	2	6.30
16	2	3	1	3	2	6.65
17	2	3	2	1	3	7.24
18	2	3	3	2	1	5.74

**Table 10:** Response table of Taguchi.

Level	Gate Type	Filling Time	Cooling Time	Pressure Holding Time	Melt Temperature
L1	7.67	5.19	6.60	5.48	7.07
L2	6.72	8.60	8.03	7.84	7.23
L3	NA	7.80	6.97	8.28	7.29
Difference	0.95	3.41	1.43	2.80	0.22

**Table 11:** Simulation result based on the optimum level of the selected parameters from Taguchi.

	Short Shot	Volume Shrinkage	Warpage	Sum
Weight	0.5	0.2	0.3	
Defects	45.03	20.94	1.26	
Normalized	0.033	0.192	0.025	***0.2499***

**Table 12:** Analysis of variance.

Factor	f	SS	PC (%)
A	1	0.009	3.59
B	2	0.109	42.8
C	2	0.016	6.15
D	2	0.069	26.66
E	2	0.002	0.89
pool error	8	0.05	20.6
Total	17	0.26	100

**Table 13:** Simulation result based on the optimum level of the selected parameters from ANN.

	Short Shot	Volume Shrinkage	Warpage	Sum
Weight	0.5	0.2	0.3	
Defects	54.66	17.96	1.01	
Normalized	0.1803	0.0658	0.0	***0.2461***

## Data Availability

The data presented in this study are available on request from the corresponding author.
